# Age- and region-specific hepatitis B prevalence in Turkey estimated using generalized linear mixed models: a systematic review

**DOI:** 10.1186/1471-2334-11-337

**Published:** 2011-12-12

**Authors:** Mehlika Toy, Fatih Oguz Önder, Tanja Wörmann, A Mithat Bozdayi, Solko W Schalm, Gerard J Borsboom, Joost van Rosmalen, Jan Hendrik Richardus, Cihan Yurdaydin

**Affiliations:** 1 Department of Public Health, Erasmus MC, University Medical Center Rotterdam, Dr. Molewaterplein 50,3000 CA Rotterdam, the Netherlands; 2 LiverDoc, Rotterdam, the Netherlands; 3 Department of Gastroenterology, Yuksek Ihtisas Hospital, Kızılay Sk. 06100 Sıhhıye, Ankara, Turkey; 4 Department of Public Health Medicine, School of Public Health, University of Bielefeld, Bielefeld, Northrhine-Westphalia, Germany; 5 Institute of Hepatology, Ankara University, Cemal Gursel caddesi, Ankara, Turkey; 6 Department of Gastroenterology and Hepatology, Erasmus MC, University Medical Center Rotterdam, Dr. Molewaterplein 50,3000 CA Rotterdam, the Netherlands; 7 Department of Gastroenterology, School of Medicine, Ankara University, Cemal Gursel caddesi, Ankara, Turkey

## Abstract

**Background:**

To provide a clear picture of the current hepatitis B situation, the authors performed a systematic review to estimate the age- and region-specific prevalence of chronic hepatitis B (CHB) in Turkey.

**Methods:**

A total of 339 studies with original data on the prevalence of hepatitis B surface antigen (HBsAg) in Turkey and published between 1999 and 2009 were identified through a search of electronic databases, by reviewing citations, and by writing to authors. After a critical assessment, the authors included 129 studies, divided into categories: 'age-specific'; 'region-specific'; and 'specific population group'. To account for the differences among the studies, a generalized linear mixed model was used to estimate the overall prevalence across all age groups and regions. For specific population groups, the authors calculated the weighted mean prevalence.

**Results:**

The estimated overall population prevalence was 4.57, 95% confidence interval (CI): 3.58, 5.76, and the estimated total number of CHB cases was about 3.3 million. The outcomes of the age-specific groups varied from 2.84, (95% CI: 2.60, 3.10) for the 0-14-yearolds to 6.36 (95% CI: 5.83, 6.90) in the 25-34-year-old group.

**Conclusion:**

There are large age-group and regional differences in CHB prevalence in Turkey, where CHB remains a serious health problem.

## Background

Globally, hepatitis B is one of the most common infectious diseases. Estimates indicate that at least 2 billion people have been infected with the hepatitis B virus (HBV), with more than 378 million people being chronic carriers (6% of the world population). Of all chronic hepatitis B (CHB) cases, approximately 40% will develop cirrhosis, liver failure, or hepatocellular carcinoma (HCC) [1,2]. According to the World Health Organization (WHO) classification, Turkey is one of the countries with intermediate (2%-8%) endemicity for hepatitis B. This information has been derived mainly from studies in blood donors. Based on these data, the overall prevalence of the hepatitis B surface antigen (HBsAg), which is a marker for chronic hepatitis B infection, has been reported to be between 4.0% and 5.0% [3], which has decreased to 2.0% in recent years [4]. However, this HBsAg prevalence appears to differ considerably in various parts of the country. For example, a study on the seroprevalence of HBV in children in Eastern Anatolia reveals an HBsAg prevalence of 9.8% [5]. There is no clear picture of the current HBV situation in Turkey. A national vaccination program for the prevention of perinatal transmission of hepatitis B infection was launched in Turkey in 1998. Currently there is no routine HBsAg screening program for pregnant women in Turkey. All infants are vaccinated at birth and followed up at 1 month and again at 6 months of age.

For the planning and implementation of adequate health promotion and intervention measures, it is important for both health care providers and policy makers to know the real burden of CHB in region- and population-specific groups. In addition, migration from Turkey to the European Union (EU) has important public health implications. Prevalence of hepatitis B in migrant populations in low endemic EU countries is likely to reflect the prevalence of their region of origin. It is of value for EU countries with Turkish migrants to know the country-specific prevalence of hepatitis B in order to make health policy decisions for migrants in their country [6]. This is particularly important for the timely identification and treatment of chronic HBV carriers. In order to make a best estimate for the age-specific, region-specific, and country-specific prevalence of HBsAg, we performed a systematic review of the literature on HBsAg prevalence in Turkey, focussing on age- and region-specific prevalence rates.

For optimal insight into the HBsAg prevalence in Turkey we included several study types: (i) studies employing random cluster sampling in the population; (ii) large-scale studies among blood donors and military conscripts; (iii) studies in various groups that have health-related concerns such as pregnant women and medical personnel; and finally (iv), studies in high-risk groups.

## Methods

### Main search strategy

This systematic review conforms to the guidelines outlined by the Meta-Analysis of Observational Studies in Epidemiology (MOOSE) [7]. For English and/or Turkish language studies, the databases MEDLINE, PUBMED, EMBASE and UlakBim (Turkish Medical Index) were searched by using the following terms: 'Hepatitis B [and] Turkey', 'HBsAg prevalence [and] Turkey'. All articles were reviewed and their corresponding reference lists inspected to identify additional material, including unpublished (grey) literature, which initially had not been detected. These were later either retrieved by a new electronic search or searched manually. The period for the meta-analysis for the age- and region-specific studies ranges from 1999-2009, so the data is coupled with results obtained after the advent of the universal immunization program in Turkey in 1999. HBsAg prevalence was estimated within 7 broad age groups (0-14, 15-24, 25-34, 35-44, 45-54, 55-64, and 65 years and older). The age groups were selected to best fit the available data extracted from the literature. For the studies on health care workers, a comparison was made between the studies from 1990-1999 and 2000-2009, in order to retrieve the risk group vaccination effect.

### Eligibility criteria

First-round review criteria for selection of studies included were the availability of explicit data on the country region, setting (e.g., hospital), study period, risk group studied, number of subjects studied, and number of subjects positive to HBsAg, or stated crude prevalence. Information related to age-specific outcome was also extracted. We took measures to detect and extract overlapping reports on the same study population. These measures included comparing the study period, sample size, centres where studies were performed, and author names.

**Figure 1:**
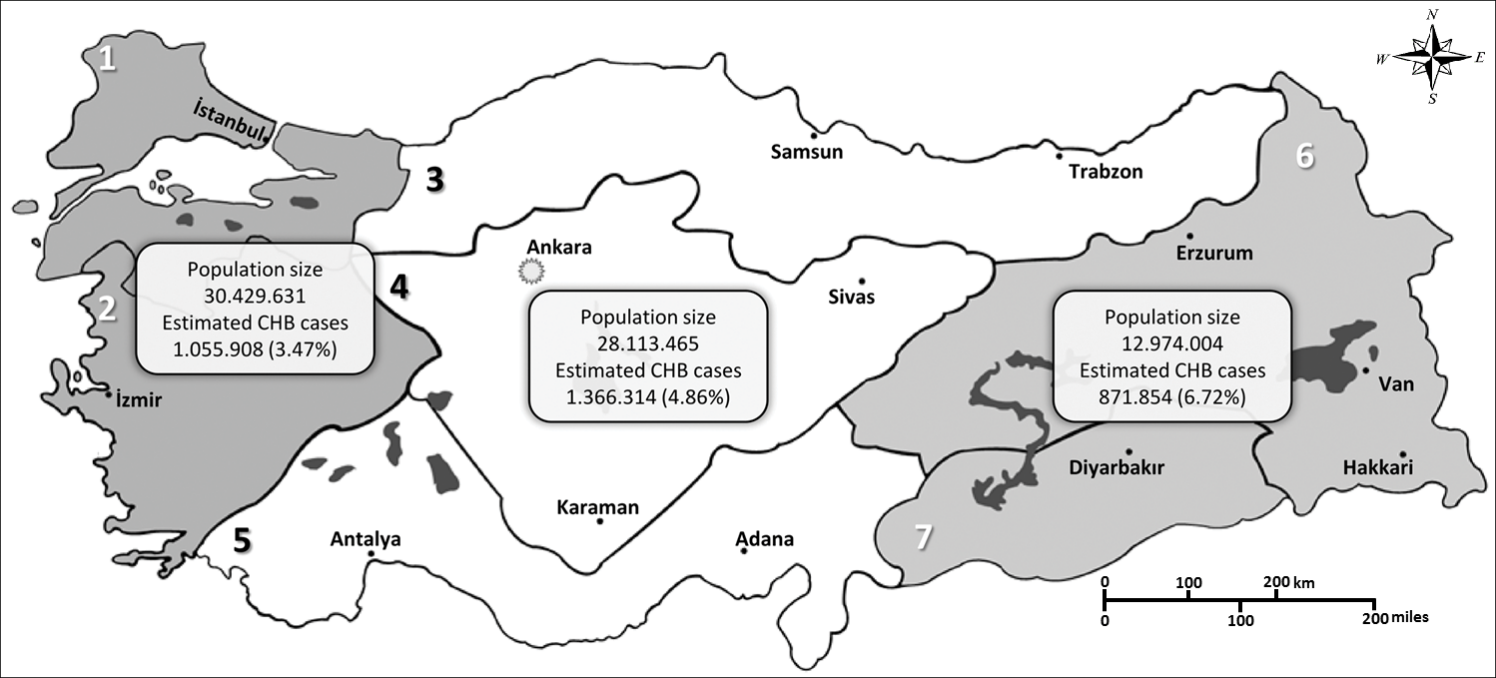
**Map of Turkey according to regions, population size per region and the number of estimated CHB cases**. Map of Turkey according to regions; 1: Marmara region, 2: Aegean region, 3: Black Sea region, 4: Inner Anatolia region, 5: Mediterranean region, 6: Eastern Anatolia region, 7: south-eastern Anatolia region. Regions with similar socioeconomic status and HBsAg seroprevalence are grouped as A (1 and 2), B (3, 4 and 5) and C (6 and 7).

The provinces of Turkey comprise 7 census-defined regions (bölge). For the purpose of this study, we pooled some regions together based on several factors such as geography, population size, and socioeconomic status. The following division of regions was done; Region A: Marmara and Aegean region; B: Black Sea, Central Anatolia and Mediterranean region; C: Eastern and south-eastern region (Figure 1).

### Data extraction and data analysis

Once a study was included, the following data were extracted and entered into tables: age group, study region, population type in terms of risk, total number of persons studied, number of persons positive for HBsAg, and year of publication. Two of the authors (MT and FOÖ) checked all data used in the analysis. When disagreement arose, these were resolved by consensus. The unpublished studies (grey literature) were included in the analysis if they met the inclusion criteria. The unpublished material consists of abstracts presented at conferences and of personal communication with clinicians who designed the sero-epidemiology studies. The names and affiliations of the authors and the settings of the unpublished studies are listed in the Additional file 1: Appendix and referred to separately from the published literature.

### Age- and region-specific randomized community sampling

The meta-analysis and data synthesis were done separately for the studies that included both region- and age-specific prevalence data, since these studies were mainly randomized community sampling. Most studies did not use a random sample from the population in each age group and sample or differed with respect to the year in which the sample was gathered. As a result, significant differences in the estimated prevalence may occur even within age groups and regions. To account for the differences among the included studies, a generalized linear mixed model was used to estimate the overall prevalence across all age groups and the 3 regions. In this model, we apply a logit link function and a binomial response distribution (similar to logistic regression) to predict the dependent variable, HBsAg positivity. The logit function is one of the most commonly used link functions for the binomial distribution.

The predictor variables of the model comprise a constant term, the categorical variables age and region, and a random intercept for each to account for unexplained variation in prevalence. The overall prevalence was estimated as the predicted value from a model that contained only a fixed intercept and a random intercept for the different studies. The model parameters were estimated using data from all studies and also separately, using only data from published studies. Model fit was evaluated from plots of the studentized residuals as a function of the linear predictor. The same method was used with region as a predictor to obtain region-specific estimates. These models were fitted with proc glimmix in SAS version 9.2 using adaptive Gaussian quadrature to approximate the log-likelihood function. From the data of these randomized community-sampling studies, we calculated the exact binomial 95% confidence interval (CI) for the crude prevalence of each study. For such CI calculations, we made the assumption that each study used a random sample from the population.

### Other studies

We calculated the weighted mean prevalence (WMP) to limit the bias caused by the heterogeneous nature of the reports. WMP was calculated as follows:WMP = ∑ωiprevi/∑ωi where ω_i _= 1/[prev_i_(1-prev_i_)/N_i_], prev_i _is the fraction of (HBsAg positive patients) in study *i*, and N_i _is the number of patients in study *i*. WMP is regarded as the most accurate method to estimate HBsAg prevalence when considering several reports [8-10]. This method has proven to be reliable when combining a number of studies with inherent heterogeneity in sample and effects size [11]. This heterogeneity can be caused by a small difference in the way that patients are sampled in the population or by differences in the year of the study. The WMP was calculated for all data (published and unpublished); in addition, for the published data separate, two-sided Mann-Whitney tests were used to compare the prevalence distribution of published and unpublished studies.

## Results

### General scope

**Figure 2:**
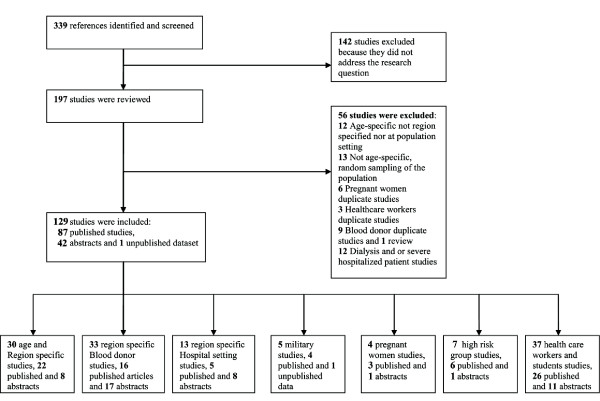
**Flow diagram (Selection Strategy) of included studies**.

The results of the search strategy and final distribution of the studies are shown in Figure 2. The electronic search identified 254 papers, and manual reference checking identified an additional 84 references; we also received 1 unpublished dataset on military recruits. Out of the 197 studies that were reviewed, 136 studies were in Turkish, of which 19 were finally excluded. The list of references arranged by various criteria can be found in Tables 1, 2, 3 &4. The systematic review identified 129 studies that provided prevalence estimates that were split into different subgroups.

**Table 1:** Age- and Region-Specific HBsAg Prevalence in Turkey, 1999-2009

Age group				**Region**^**a**^											
**(yr)**		**A**					**B**					**C**			

	**Ref**	**N**	**n**	**%HBsAg**	**95% CI**	**Ref**	**N**	**n**	**%HBsAg**	**95% CI**	**Ref**	**N**	**n**	**%HBsAg**	**95% CI**

0-14	a	420	14	3.33	1.83, 5.52	[12]	460	7	1.52	0.61, 3.11	[13]	402	13	3.23	1.73, 5.47

	b	249	11	4.41	2.22, 7.76	[14]	875	26	2.97	1.95, 4.32	c	158	9	5.70	2.64, 10.54

	d	230	6	2.60	0.96, 5.59	[15]	1131	80	7.07	5.64, 8.73	[16]	853	84	9.84	7.93, 12.04

	e	1952	67	3.43	2.66, 4.34	f	1278	22	1.72	1.08. 2.59	[17]	700	29	4.14	2.79, 5.90

	[18]	176	3	1.70	0.35, 4.90	[19]	420	13	3.10	1.66, 5.23	[20]	74	6	8.10	3.03, 16.81

	[21]	6788	84	1.24	0.99, 1.53	g	100	2	2.00	0.24, 7.03	[22]	802	22	2.71	2.45, 2.97

	[23]	423	8	1.89	0.82, 3.69	[24]	454	4	0.88	0.24, 2.24					

Total		10238	193	1.88	1.63, 2.16		4718	154	3.26	2.77, 3.81		2989	163	5.45	4.66, 6.32

**Total overall**^**b**^		**17945**	**510**	**3.23**	**2.96, 3.53**										

15-24	[25]	129	6	4.65	1.72, 9.85	[12]	1058	43	4.06	2.96, 5.44	[13]	183	7	3.82	1.55, 7.72

	[18]	440	46	10.45	7.75, 13.70	[26]	238	12	5.04	2.63, 8.64	[27]	213	46	21.60	16.27, 27-73

	[21]	2822	178	6.31	5.44, 7.27	f	2431	114	4.69	3.88, 5.60	[20]	187	29	15.50	10.63, 21.50

	[28]	7237	98	1.35	1.10, 1.65	[29]	350	11	3.14	1.58, 5.55	h	96	3	3.21	0.65, 8.86

						[30]	155	20	13.00	8.06, 19.22					

						[24]	193	7	3.62	1.47, 7.32					

						[31]	340	22	6.47	4.09, 9.63					

Total		10628	328	3.08	2.76, 3.43		4765	229	4.81	4.21, 5.45		679	85	12.51	10.12, 15.24

**Total overall**^**b**^		**15636**	**617**	**5.77**	**5.26, 6.32**										

25-34	[18]	226	18	7.97	4.79, 12.29	[12]	833	53	6.36	4.80, 8.24	[13]	219	19	8.67	5.30, 13.21

	[21]	3674	235	6.40	5.62, 7.24	f	2431	114	4.69	3.88, 5.60	[27]	324	60	18.50	14.43, 23.18

						[24]	193	7	3.62	1.47, 7.33	h	435	26	5.98	3.94, 8.63

						[31]	238	12	5.04	2.63, 8.64					

Total		3900	253	6.49	5.73, 7.30		3695	186	5.03	4.35, 5.78		978	105	10.73	8.86, 12.85

**Total overall**^**b**^		**7950**	**506**	**7.08**	**6.47-7.73**										

35-44	[18]	142	7	4.93	2.00, 9.89	[32]	85	10	11.76	5.79, 20.57	[13]	17	2	11.77	1.46, 36.44

	[21]	4323	249	5.76	5.08, 6.49	[12]	741	71	9.58	7.55, 11.93	[27]	271	41	15.13	11.08, 19.96

						f	3208	170	5.30	4.55, 6.13	h	555	38	6.84	4.90, 9.28

						[24]	193	7	3.63	1.47, 7.33					

						[31]	173	7	4.05	1.64, 8.16					

Total		4465	256	5.73	5.07, 6.45		4400	265	6.02	5.33, 6.76		843	81	9.60	7.70, 11.80

**Total overall**^**b**^		**8980**	**557**	**6.93**	**6.32, 7.60**										

45-54	[18]	85	7	8.23	3.37, 16.23	[32]	55	5	9.09	3.02, 19.95	[13]	17	2	11.76	1.46, 36.44

	[21]	2170	100	4.60	3.76, 5.58	f	3208	170	5.30	4.55, 6.13	[27]	244	29	11.88	8.10, 16.62

						[12]	288	27	9.37	6.27, 13.35	h	469	34	7.24	5.07, 9.98

						[24]	346	12	3.47	1.80, 5.98					

						[31]	143	5	3.50	1.14, 7.97					

Total		2255	107	4.75	3.90, 5.70		4040	219	5.42	4.74, 6.16		730	65	8.90	6.94, 11.20

**Total overall‡**		**6468**	**357**	**6.13**	**5.48, 6.85**										

55-64	[18]	32	0	0	0	f	4031	129	3.20	2.68, 3.79	[13]	17	2	11.76	1.46, 36.44

	[21]	631	32	5.07	3.49, 7.08	[12]	56	1	1.78	0.05, 9.55	[27]	125	15	12.00	6.87, 19.01

						[24]	346	12	3.47	1.80, 5.98	h	463	30	6.48	4.41, 9.12

						[31]	118	11	9.32	4.75, 16.06					

Total		663	32	4.83	3.32, 6.74		4551	153	3.36	2.85, 3.93		605	47	7.77	5.76, 10.19

**Total overall**^**b**^		**5238**	**191**	**5.03**	**4.29, 5.89**										

65+	[33]	165	15	9.09	5.17, 14.55	f	204	5	2.45	0.80, 5.63	[13]	17	2	11.76	1.46, 36.44

	[18]	89	2	2.24	0.27, 7.88	[24]	714	29	4.06	2.74, 5.78	[27]	127	10	7.87	3.84, 14.00

	[21]	1115	35	3.14	2.20, 4.34	[31]	83	3	3.61	0.75, 10.20	h	1140	28	2.45	1.64, 3.53

Total		1369	52	3.80	2.84, 4.95		1001	37	3.70	2.61, 5.06		1284	40	3.11	2.23, 4.21

**Total overall**^**b**^		**2431**	**98**	**3.52**	**2.95-4.19**										

**Overall Prevalence**^**c**^				**4.57**	**3.58, 5.76**										

Abbreviations: *N *number of subjects, *n *number of cases, *Ref *reference

^a ^Region A: Marmara and Aegean, B: Black Sea, Central Anatolia and Mediterranean, C: Eastern and south-eastern

^b ^Total number of persons, cases and the pooled prevalence for each age-group

^c ^The overall prevalence over all age-groups and the three regions

**Table 2:** Weighted mean region-specific HBsAg prevalence in blood donors and hospital settings

Study group					%HBsAg prevalence (range)			
**Category**	**no.studies**	**N**	**n**	Region*	**Weighted mean all data**	**Weighted mean published data**	***P *value**	**References**

Blood donors	13	658662	16788	A	2.53 (1.10, 8.70)	2.33 (1.70, 2.75)	0.51	[34-40] (i-n)

	16	223949	6389	B	2.68 (1.70, 4.22)	3.44 (2.60, 4.20)	0.14	[41-46] (o-x)

	4	149918	6675	C	4.25 (1.70, 4.90)	4.45 (2.92, 4.90)	0.50	[47-49] (y)

Hospital†	5	37497	2977	A	3.40 (1.30, 13.80)	1.30 (0.90, 1.63)	0.40	[50] (z-cc)

	6	100343	7781	B	7.15 (2.90, 13.60)	5.73 (2.90, 13.60)	1.00	[51,52] (dd-gg)

	2	28392	6037	C	-	10.88 (9.60, 21.30)	-	[53,54]

Abbreviations: *N *number of subjects, *n *number of cases

*P *value calculated by the two-sided Mann-Whitney test (difference in prevalence of all data and only published data)

* Region A: Marmara and Aegean, B: Black Sea, Central Anatolia and Mediterranean, C: Eastern and South-eastern

† Studies based in hospital settings on non-liver-related conditions

**Table 3:** Weighted mean HBsAg prevalence in the military and in pregnant women

Study group				%HBsAg prevalence (range)			
**Category**	**no.studies**	**N**	**n**	**Weighted mean all data**	**Weighted mean published data**	***P *value**	**Reference**

Pregnant Women	4	6215	137	1.70 (1.31, 9.30)	1.60 (1.31, 3.50)	0.50	[55-57] (hh)

Military	5	90914	3539	3.60 (2.16, 9.80)	3.83 (2.16, 9.80)	0.80	[58-61] (ii)

*P *value calculated by the two-sided Mann-Whitney test (difference in prevalence of all data and only published data)

**Table 4:** Weighted mean HBsAg prevalence rates of health care workers and students before and after vaccination

Risk-group					%HBsAg prevalence (range)			
**Category**	**year**	**no.studies**	**N**	**n**	**Weighted mean all data**	**Weighted mean published data**	***P *value**	**Reference**

HCW	1989-1999	15	4147	191	3.34 (1.60, 8.60)	3.86 (1.90, 8.00)	0.97	[62-74] (jj-kk)

HCW	2000-2009	15	5146	137	2.29 (1.40, 5.90)	2.35 (1.40, 5.90)	0.60	[75-83] (ll-qq)

HCS	1989-1999	4	1022	39	3.31 (2.40, 8.60)	3.42 (2.60, 8.60)	0.50	[84-86] (rr)

HCS	2000-2009	3	438	8	2.08 (1.00-2.40)	2.00 (0.20, 3.70)	1.00	[87] (ss-tt)

Abbreviations: *N *number of subjects, *n *number of cases, *HCW *health care worker, *HCS *health care student

*P *value calculated by the two-sided Mann-Whitney test (difference in prevalence of all data and only published data).

### Age- and region-specific studies

**Figure 3:**
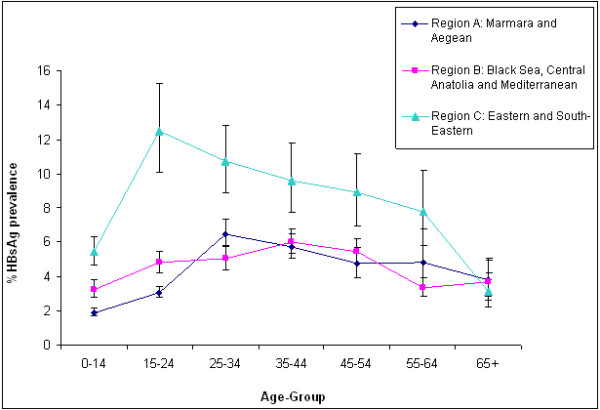
**Age- and region-specific HBsAg prevalence, 1999-2009**.

Thirty studies, of which 22 were published [12-33] and 8 unpublished (Additional file 1: Appendix; a-h), reported the age- and region-specific prevalence of hepatitis B. Both the age- and region-specific prevalence and the overall population prevalence are shown in Table 1. The overall age group prevalence for regions A, B, and C yields 3.52 (95% CI: 1.92, 5.11), 4.95 (95% CI: 3.06, 6.83), and 6.76 (95% CI: 4.58, 8.93), respectively. Figure 3 shows the age group and region-specific curves of the pooled prevalence data. The estimated overall population prevalence was 4.57% (95% CI 3.58, 5.76). The generalized linear mixed model was used to also estimate the prevalence using only the published data, which yielded a population prevalence of 5.10 (95% CI: 4.22, 6.34). When this prevalence is extrapolated to the total population living in Turkey (71.5 million), the total number of CHB cases will be about 3.3 million (Figure 1). We included the year of study as a linear term in the model, which turned out to be insignificant with a *P *value of 0.07.

### Blood donors and hospital setting (non-donor population) by region

A total of 33 blood donor studies, of which 16 were published [34-49] and 17 unpublished (Additional file 1: Appendix; i-y). A total of 13 hospital-setting studies were identified, of which 5 were published [50-54] and 8 unpublished (Additional file 1: Appendix; z-gg), separated into region-specific outcomes. The weighted mean prevalence (WMP) of HBsAg increases from region A to C, from 2.53 to 4.25, respectively. The same trend is seen in the weighted mean prevalence calculations in the hospital settings. For both study groups, there were fewer studies in region C than in the other 2 regions (Table 2).

### Pregnant women and military conscripts (age range, 17-41 years)

The results for pregnant women [55-57] and military recruits [58-61] are presented in Table 3. Military service is compulsory in Turkey for men between the ages of 17 and 39 years. The age range in the studies of pregnant women varied widely, from 17 to 41 years, which is equivalent in range to that of the military recruits, which is why we put these 2 study populations in the same table. From the results of the WMP, we observed a gender difference, which is 3.60% for military recruits and 1.70% for pregnant women.

### Health care workers and students

As there have been vaccination policies for health care workers in Turkey since 1999, we separated the results into 2 sections. We compared the prevalence rates of studies between 1990 and 1999, and 2000 and 2009 for both health care workers (HCW) [62-83] and health care students (HCS) [84-87]. Table 4 shows the decrease in prevalence within the 10-year periods before and after vaccination for HCW and HCS; the WMP decreased from 3.34 to 2.29, and from 3.31 to 2.08, respectively.

### High-risk groups

To show the prevalence among those at high risk for hepatitis B, we present the results for the following groups: high-risk occupations; prison inmates; and female sex workers. The high-risk occupation group consisted of studies among hairdressers, butchers, garbage men, and coffee house workers. There were 2 studies [88,89] that were eligible and used to calculate the WMP, which was 7.63. There were 3 prison inmate studies, which were all unpublished (uu-ww) and yielded a WMP of 6.73. Two studies, of which 1 was published [90] and the other unpublished (Additional file 1: Appendix; xx), were analyzed for the female sex worker (FSW) group, which yielded a WMP of 7.00.

## Discussion

### Age- and region-specific prevalence estimates

The age- and region-specific analysis relied primarily on community-based studies from various regions. The results yielded a marked difference in the overall region-specific estimated prevalence, which ranged from 3.52 to 6.76. The age-specific prevalence also varied greatly between the lowest prevalence in the age group of 0-14 years (2.84) and the highest prevalence, age group 25-34 years (6.36). The overall country-specific prevalence was 4.57, retrieved from the meta-analysis, across all regions and age groups.

### Region-specific, HBsAg-prevalence estimates for different sample populations

Population sample groups such as blood donors have been used as a convenient means to estimate the country-specific prevalence of HBV [4]. Since blood donation in Turkey is voluntary and the predonation eligibility assessment is quite strict, a healthy donor effect prevails in this sample population, and likely leads to an underestimation of the true HBV prevalence. Blood donors in this case cannot be representative of an entire population. Our results showed a difference in the region-specific estimates, where the weighted mean prevalence (WMP) ranges from 2.53 to 4.25. When compared with the age- and region-specific outcomes, the results in this sample population are in the low range, whereas the results in the hospital-setting studies are in the high range. Thus, results based on blood donors and from hospital settings should be interpreted with caution.

Another convenient sample of population-based studies is the military conscripts. Military service prevalence studies provide good estimates for the general population of men between the ages of 17 and 41, while pregnant women studies represent the comparable female population of the same age range. Both these sample groups are more representative than blood donors and those from hospital settings. Although screening for HBV in pregnant women is not yet routine in Turkey, nonetheless pregnant women are likely to be tested during their pregnancies, either for research or for health purposes. Our WMP results comparing these 2 groups suggest that HBV is more prevalent among males.

### Strengths and limitations

The main strength of this systematic review is that it includes all available Turkish studies, including both published and unpublished abstracts (grey literature), to overcome publication bias, and in particular language bias. Due to the paucity of studies from Turkey on hepatitis B in English, this review provides a wealth of information that would not be accessible to scientists and policy makers from other countries in the world. We used an innovative approach to fit generalized linear mixed models in estimating the prevalence from various studies. Including the year of the study as a linear term in the model showed no significant trend in prevalence over time. Since prevalence is assumed to be not constant, this could be a limitation of the model. Another limitation may be the dependence on the quality of the original reports. The strength of the study may also be a weakness in that conventional wisdom points towards an inverse correlation between quantity and quality. Despite this limitation, we believe that the study provides useful data on the epidemiology of hepatitis B in Turkey for health planning strategies, both in Turkey as well as in the Turkish migrant population. We suggest that researchers who are preparing observational research, such as sero-survey studies, implement the STROBE guidelines [91] to ensure a clear presentation of what was planned, done, and found in such a study.

### Comparing the eras of pre-vaccination and post-vaccination

Since the implementation of universal vaccination in 1998 of all children and risk groups, a decline in prevalence has been observed [4]. Although the current study does not address this issue directly, the availability of age-specific prevalence rates in the postvaccination era enables us to make meaningful comparisons in children from studies in the prevaccination era. In this context, Kanra et al. [92] studied the prevalence in all regions of Turkey among children before the vaccination policy was implemented. Their finding of an overall country prevalence of 5.90 among 0-15-year-olds compares favourably to the current overall country prevalence rate of 2.84 reported in this study for the same age group. The impact of vaccination was also assessed in health care workers and health care students. The WMP estimates in the postvaccination studies show a decline, which could be explained by the impact of the vaccination campaign or, as a secondary explanation, that HBV has the tendency to decrease over the years. A study from the United States shows patterns in the success of vaccination application to health care workers [93].

### Implications for health policy

Nearly every country with a large or diverse geographic area is expected to have regional differences in HBV prevalence, and the extent of the geographic variation can be very important. The large regional differences in prevalence in Turkey are mainly due to differences in socioeconomic status, lifestyles, infrastructure, and access to health services. In the eastern and south-eastern regions (treated as 1 region) of Turkey, all reasons mentioned above apply in a negative way, though the latest years have witnessed much improvement in the socioeconomic, and hygienic and sanitary conditions in this region and in Turkey in general. This region also lags behind in coverage of HBV vaccination. Although only 18% of the total population live in this area, the estimated number of CHB cases is almost equal to that of the other regions, which have higher population numbers (see Figure 1). A substantial migration has taken place from the southeast and east to the west of the country, mainly for economic reasons. The scarcity of reports from southeast and east Turkey, despite the magnitude of the CHB problem, may be an indirect reflection of the health infrastructure of this region. The region-specific data in this study could stimulate a broad-based prevention and control campaign whereby hepatitis B vaccines and/or treatment and monitoring could be targeted to high-priority regions. Turkey has a large proportion of young people (age 14-30 years, which is more than 66% of the total population). It is a dynamic society with a growing number of educated people; further, the proportion of the population living in cities has increased dramatically in recent decades and now accounts for approximately 70% of the national total. With an average prevalence of 3.50 in young people age 0-24 years, hepatitis B remains a significant public health problem in Turkey.

Another important facet of the data in this study is linked to disease awareness, although this is certainly not specific to Turkey, since CHB patients in general are mostly asymptomatic. With an overall HBV prevalence of 4.57, the estimated number of HBV carriers in Turkey is 3.3 million. Even a very conservative assessment means that 10% of the carriers would need treatment, yielding 330,000 chronic HBV cases eligible for treatment in Turkey alone. We recently estimated that treatment of CHB patients with active disease with a low-resistance profile drug could reduce mortality related to liver disease in this group by 80% [94]. It needs to be stressed that in Turkey, viral hepatitis treatment is fully reimbursed through the national insurance system. According to net sold medication counts per year, it was calculated that no more than 10% of eligible patients receive active treatment [95], indicating a massive shortcoming in ensuring prolonged life and even life-saving treatments. Chronic HBV infection is a lifelong illness. It can cause serious, life-threatening complications, such as cirrhosis, liver cancer, or liver failure. Most liver transplants in Turkey are attributed to liver disease from chronic HBV. A viral hepatitis national plan should be designed that will lower hepatitis prevalence, increase research, and accelerate access to care for the chronically infected. Testing for immunization coverage should be instituted at the state and regional level. Potential next steps would be to improve epidemiologic surveillance systems, develop a hepatitis registry, and implement serosurveys in order to produce reliable data to guide prevention and control measures and to monitor the impact of preventive strategies.

The importance of our study is certainly not confined to the borders of Turkey. Recent evidence suggests that the overall decline in HBV prevalence in the last decade in industrialized countries of Europe appears to have reached a plateau. The most likely reason why the progressive decline in HBV prevalence has come to a halt is migration from endemic areas [96,97]. There are currently more than 3 million immigrants, descendants of immigrants, and naturalized citizens and political refugees from Turkey in Western Europe, representing the largest immigrant group in the European Union. The public health implications of the current study thus go far beyond the border of Turkey.

## Conclusions

Despite the availability of a safe and effective vaccine for more than 20 years and because of its asymptomatic nature, CHB remains a serious health problem. As this study shows, the estimates of age- and region-specific prevalence reflect the burden of existing CHB cases in Turkey. Knowing the diversity in prevalence of CHB in Turkey, public health organizations should turn their attention, means, and actions increasingly to the areas and groups that are lagging behind.

## List of abbreviations

CI: Confidence interval; CHB: Chronic hepatitis B; EU: European Union; HBsAg: Hepatitis B surface antigen; HBV: Hepatitis B virus; HCC: Hepatocellular carcinoma; HCS: Health care worker; HCW: Health care student; WHO: World Health Organization; WMP: Weighted mean prevalence.

## Competing interests

The authors declare that they have no competing interests.

## Authors' contributions

MT designed the study, collected the data, and wrote the manuscript. FOÖ participated in data collection, literature review, and writing of the manuscript. TW participated in the literature review and prepared the Tables and Figures. AMB supervised the literature review. SWS assisted with the overall study design and the writing of the manuscript. GJB built the generalized linear mixed models. JvR supervised the statistical analysis and helped in preparing Tables and Figures. JHR supervised the public health aspects of the study and assisted in the preparation of the manuscript. CY was responsible for the overall supervision of the study. All authors have read and approved the manuscript.

## Pre-publication history

The pre-publication history for this paper can be accessed here:

http://www.biomedcentral.com/1471-2334/11/337/prepub

## Supplementary Material

Additional file 1 **Appendix: grey literature (DOC 27 kb)**. Click here for file
