# Supplementary material for: Age- and region-specific hepatitis B prevalence in Turkey estimated using generalized linear mixed models: a systematic review
Source: BMC Infect Dis. 2011 Dec 12;11:337. doi: 10.1186/1471-2334-11-337 (PMC3262158; doi:10.1186/1471-2334-11-337)
Supplement: Additional file 1 — Appendix: grey literature (DOC 27 kb). [file 1471-2334-11-337-S1.DOC]

**Appendix**

Unpublished (grey) literature:

1. Tosun- Yegane S, Bilge B, Önder U, Gündüz T, Ertekin E. HBV ile karşılaşma olasılığının sosyo-ekonomik koşullarla ilişkisi (Relation of HBV exposure risk and socioeconomic status). 5th National Viral Hepatitis Congress Abstract Book. 2000:P-B18.
2. Hacımustafaoğlu M, Çelebi S, Sadıkoğlu G. Çocuklarda Hepatit B prevalansı. (Hepatitis B prevalance in children). 4th Pediatric Gastroenterology and Nutrition Congress Abstract Book. 2000:249.
3. Aslan G, Tatlı M, Ulukanlıgil M, Seyrek A, Ataş A. Şanlıurfa ilinde bebeklerde HBV seroprevelansı (Hepatitis B seroprevalence in infants in Şanlıurfa). 5th National Viral Hepatitis Congress Abstract Book. 2000:P-B30.
4. Şahin S, Kardeşler S. Kırklareli’nde çocukluk yaş grubunda hepatit B seroprevalansı ve aşılama durumu. (Seroprevalance of hepatitis B and vaccination status of children in Kırklareli). 1st Pediatric Infectious Disease Congress Abstract Book. 1999:284.
5. Zülfikar B, Öztürk A, Kınık K. İstanbul’da değişik yaş grubundan sağlıklı kişiler, hamileler, diş hekimleri ve berberlerde hepatit B taraması sonuçları (Results in screening of barbers, dentists, pregnant women and healthy volunteers in various age groups for hepatitis B in İstanbul). Viral Hepatitis Symposium Abstract Book. 1996:20.
6. Yousefi Radi A, Bingöl N, Aslantük A, Demirboğa S. Çeşitli yaş gruplarında HBsAg ve Anti-HBs prevalansı. (HBsAg and Anti-HBs prevalance in various age groups) 9th National Clinical Microbiology and Infectious Diseases Congress Abstract Book. 1999:187.
7. Atabek M, Ural O, Çoban H, Karaeren Z, Aydın K, Erkul I. Bölgemizde çocukluk ve erişkin yaş gruplarında hepatit A,B ve C belirleyicilerinin araştırılması (Investigaton of hepatitis A,B and C markers in children and adults in our region). 1st Pediatric Infectious Disease Congress Abstract Book. 1999:272.
8. Karadag M, Erturk M. Gaziantep Avukat Cengiz Gökçek Hastanesinde Hepatit B ve C sıklığı (Hepatitis B and C prevalence in Avukat Cengiz Gokcek hospital in Gaziantep). National Gastroenterology Conference. 2009:P370.
9. Gültekin B, Eyigör M, Yaman S, Aydın N. Adnan Menderes Ü Tıp Fakültesi 2001 yılında hasta ve donörlerde HbsAg prevelansı (Prevalence HBsAg in patients and blood donors applied to Adnan Menderes University Hospital in 2001). 6th National Viral Hepatitis Congress Abstract Book. 2002:52.
10. Göz S, Demirel A, Gez A, Akdağ B. Kan ve afarez donörlerine serolojik bakış. (Serologic tests for blood and apheresis donors). 7th National Viral Hepatitis Congress Abstract Book 2004:127.
11. Hanedan S, Ayaz N, Öztürk S. Kan Merkezimize başvuran donörlerin donör tarama testleri verileri. (Results of donor screening tests applied to blood bank) 19th Microbiology Congress Abstract Book 2000:358.
12. Küçükateş E. İstanbul Üniversitesi Kardiyoloji Enstitüsü Kan Donörlerinde HBsAg taraması. (HBsAg screening of blood donors applied to İstanbul University Cardiology Institude Blood Bank). 6th National Viral Hepatitis Congress Abstract Book. 2002:38.
13. Küçükateş E, Bilgen H. İ.Ü.Cerrahpaşa kan merkezi hepatit B ve C taraması. (Results of Screening for Hepatitis B and C in İ.U. Cerrahpasa Blood Bank). 7th National Viral Hepatitis Congress Abstract Book. [Abstract book]. 2004:97.
14. Olut A, Köse Ş, Kula A, İyi T. Tepecik SSK Eğitim ve Araştırma Hastanesi Kan Merkezindeki donörlerde hepatit B ve C seroprevalansı (Seroprevalence of hepatitis B and C in donors in blood donors Tepecik Hospital). 7th National Viral Hepatitis Congress Abstract Book. 2004:112.
15. Kaya A, Demirci M, Cicioğlu-Arıdoğan B, Adiloğlu A. Isparta Bölgesi kan donörlerinde HbsAg ve anti-HCV prevelansı. (HBsAg and anti-HCV prevalance in blood donors in Isparta region). 30th Turkish Clinical Microbiology Congress Abstract Book. 2002:305.
16. Tezeren D, Aksaray S, Töyran A, Güvener E. Ankara Numune Eğitim ve Araştırma Hastanesine başvuran kan donörlerinde HbsAg ve Anti-HCV prevelansı (The prevalence rate of HBsAg and anti-HCV in blood donors applied to Ankara Numune Trainig and Research Hospital). 7th National Viral Hepatitis Congress Abstract Book. 2004:117.
17. Uraz G, Türkmen L, Tulumoğlu Ş. Çankırı yöresinde 1953 donörde HbsAg ve anti-HCV sıklığı.(Prevalence of HBsAg and anti-HCV in 1953 donors in Çankırı region). 29th Turkish Microbiology Congress Abstract Book. 2000:397.
18. Türkmen L. 8500 donörde HbsAg ve anti-HCV sonuçlarının retrospektif değerlendirilmesi (Retrospective analysis of HBsAg and anti-HCV results of 8500 blood donors). 10th KLİMİK Congress Abstract Book. 2001:341.
19. Yavuz T, Behçet M, Şahin İ, Öztürk E, Özdemir D. AİBÜ Düzce Tıp Fakültesi Kan Merkezine başvuran donörlerde HBV, HCV, HIV ve sifiliz seroprevalansı. (Seroprevalence of HBV,HCV,HIV and syphilisis in blood donors appied to AIB University Duzce School of Medicine). 2nd National Virology Congress Abstract Book. 2005:296
20. Ünlü P, İynem H, Özyıldırım S. Karabük ilinde HbsAg ve anti-HCV seroprevalansı. (Seroprevalence of HBsAg and anti-HCV in Karabuk). 2nd National Virology Congress Abstract Book. 2005:284.
21. Öztürk C, Delialioğlu N. Mersin Ü Tıp Fakültesi Araştırma ve Uygulama Hastanesi Kan merkezinin 15 aylık verilerinin değerlendirilmesi. (Evaluation of 15 months data of blood bank of Mersin University Hospital). 29th Turkish Microbiology Congress Abstract Book. 2000:356.
22. Otağ F, Köksel T. Mersin Ü Tıp Fakültesi Araştırma ve Uygulama Hastanesi Kan merkezi donörlerinin enfeksiyon etkenleri tarama sonuçlarının değerlendirilmesi (Evaluation of results of infectious disease screening tests of donors applied to Merisn University). 6th National Viral Hepatitis Congress Abstract Book. 2002:81.
23. Tiftik N, Ünal T, Kırık P, Yalçın A. The results of microbiologic screening test in blood donors in Mersin Turkey. 30th World Congress of International Society of Hematology Abstract book. 2005:318.
24. Kaya S, Poyraz Ö, Sümer Z, Özdemir L. Cumhuriyet Üniversitesi Tıp Fakültesi Hastanesi Kan Merkezine Başvuran Donörlerde saptanan infeksiyon etkenlerinin 5 yıllık peryotta retrospektif olarak değerlendirilmesi. (Retrospective analysis of prevelance of infection markers in donors applied to Cumhuriyet University blood bank in 5 years) 7th National Viral Hepatitis Congress Abstract Book. 2004:116.
25. Özden M, Denk A, Almış H, Kılıç S. Kan donörlerinde HbsAg ve anti-HCV seropozitifliği. (Seropositivity of HBsAg and anti-HCV in blood donors). 7th National Viral Hepatitis Congress Abstract Book. 2004:126.
26. Arabacı E, Oldacay M. Çanakkale Devlet Hastanesi Merkez Laboratuarı HBV, HCV ve HIV sonuçları (HBV,HCV and HIV test results of Canakkale Government Hospital). 7th National Viral Hepatitis Congress Abstract Book. 2004:125.
27. Biçmen C, Eriş N, Şenol G, Florat N. İzmir Göğüs Hastalıkları ve Cerrahisi Eğitim Hastanesinde yatan hastalarda HBV, HCV ve HIV seroprevalansı (Seroprevalence of HBV,HCV and HIV in patients hospitalized in İzmir Pulmonary Disease and Chest Surgery Hospital). 29th Turkish Microbiology Congress Abstract Book. 2000:357.
28. Köse Ş, Özkalay N, Avcıbaşı P, Gürsoy G, Çamcı G. SSK Tepecik Eğitim ve Araştırma Hastanesinde Hepatit Belirteçlerinin Seroprevalansı (Seroprevalence of hepatitis markers in Tepecik Hospital). 7th National Viral Hepatitis Congress Abstract Book. 2004:123.
29. Terek G, Çoban H, Ağca H, Özbek Ö. Preoperatif dönemdeki olgularda HBsAg, anti-HCV, HIV-1 ve RPR pozitiflik oranları (HBsAg, anti-HCV, HIV-1&2 and RPR positivity rates in cases in preoperative period). 2nd National Virology Congress Abstract Book. 2005:289.
30. Özen M, Ergönül Ö, Balaban N. Ankara Numune Eğitim ve Araştırma Hastanesi ELISA laboratuarında saptanan hepatit A, B, C seroprevalansı değerleri. (Seroprevalence data of hepatitis A, B and C viruses of ELISA laboratory of Ankara Numune Hospital) 5th National Viral Hepatitis Congress Astract Book. 2000.
31. Battal I, Kurt H, Tekeli E. Ankara’da sağlıklı bireylerde HAV, HBV ve HCV seropozitifliğinin yaş ve cinsiyete göre dağılımı. (Age and gender specific distrution of HAV,HBV and HCV seroprevalanece in Ankara). 5th National Viral Hepatitis Congress Abstract Book. 2000:P-B14.
32. Bilgili H, Değirmenci S, Tezeren D, Aksaray S, Güvener E. Ocak 2001 – Mayıs 2002 döneminde Ankara Numune Eğitim ve Araştırma Hastanesi ELISA laboratuarında saptanan HBV ve HCV seroprevalansı (Seroprevalence of HBV and HCV detected in Ankara Numune Hospital between January 2001 and May 2002). 30th Turkish Clinical Microbiology Congress Abstract Book. 2002:305.
33. Öztürk E, Ağartan C, Kaya D, Şahin İ, Şencan İ, Ertör O. Düzce’de Hepatit Göstergelerinin İki Yıllık İnceleme Sonuçları (Hepatit Prevalence in Duzce – Results Of Two Years Period). 6th Naitonal Viral Hepatitis Congress Abstract Book. 2000:82.
34. Sırmatel F, Bozkurt A, Karslıgil İ, Özçırpıcı B, Özgür S. Gebelerde hepatit B araştırması ve HBsAg (+) annelerinde doğumdan sonra aşılanan çocuklarının hepatit yönünden izlem sonuçları (Prevalence of HBV in pregnant women and results of HBV vaccination of children born from HBsAg positive mother). 6th National Viral Hepatitis Congress Abstract Book. 2002:47.
35. Kocak N. Yirmi Yas komando askerlerde bolgelere gore yillik HBsAg oranlari. *Unpublished data*.
36. Aydin M, Avlak U, Unlu S. Dr. Zekai Tahir Bulak arastirma hastanesi personelinde hepatit B seroprevalenasi (Hepatitis B prevalence among medical personnel in Dr.Zekai Tahir Bulak research hospital). Viral hepatit sempozyum program ve kongre kitabi Ankara. 1996:s22.
37. Sunbul M, Sanic A, Eroglu C, Akcam Z, Hokelek M, Leblebicioglu H. Hastane personeli 645 kiside HbsAg, anti-HBs ve anti-HBc tarama sonuclari (HBsAg, anti-HBs and anti-HBc screening among 645 medical personnel). Turk klinik mikrobiyoloji ve enfeksiyon hastaliklari kongresi kitabi. 1997:s427.
38. Firinciogullari F, Aksoy N, Candemir A. Cukurova U.Tip fakultesi hastanesi saglik calisanlarinda hepatit B imunizasyon durumu (Hepatitis B immunization among medical pesonnel in Cukurova university and medical center hospital). Ulusal Viral hepatit kongresi kitabi 2004:111.
39. Eldemir H, Demirturk N, Kesli R. SSK Afyon Hastanesi personelide HB ve HC seroprevalansi (HBV and HCA seroprevalence among medical personel in SSK Afyon hospital). Ulusal hepatit kongreso kongre kitabi,Viral hepatit Savasim dernegi. 2002:73.
40. Tevfik-Yavuz M. Izzet Baysal Bolu devlet hastanesi personelinde hepatit B ve C seroprevalansi (Hepatitis B and C seroprevalence among medical personnel in Izzet Baysal Bolu state hospital). Turk klinik mikrobiyoloji ve infeksiyon hastaliklari Kongre Kitabi. 2001:343.
41. Karabay O, Tamer A, Tabtaci M, Celebi H. Saglik calisanlarinin HBV gostergelerinin ve asilama duzeyinin saglik personeli olmayanlarla karsilastirilmasi (Comparison of HBV infection and vaccination among health and non-health care workers). Viral hepatit kongresi kongre kitabi viral hepatit savasim dernegi. 2004:115.
42. Kocak F, Simsek S. Saglik calisanlarinda HBV bagisikliginin degerlendirilmesi (HBV immunity among medical personnel). Ulusal viral hepatit kongresi kongre kitabi, viral hepatit le savasim dernegi. 2002:42.
43. Akgul S, Gunduz T, Borand H, Ispir B, Avci-otnu A. Hastane personellerinin hepatit B hakkinda bilgi duzeyleri ve HBV nin serolojik markirlarinin arastirilmasi (HBV serologic maker and HBV information knowledge among hospital personnel). Ulusal viral hepatit kongresi kongre kitabi, viral hepatit le savasim dernegi. 2004:86.
44. Aktas AE, Yigit N, Ayyildiz A, Babacan M. Tip fakultesi 3. sinif ogrencilerinin HAV, HBV, HCV enfeksiyonu ile karsilasma oranlari (The risk of HAV, HBV and HCV infection among 3rd year medical students) Turk klinik mikrobiyoloji ve infeksiyon hastaliklari kongresi kongre kitabi. 1999:s237.
45. Memis S, Turk G. Saglik yuksekokulu 1. sinif ogrencilerin hepatit B seroprevalansi ve risk faktorleri (Hepatitis B seroprevalence and risk factors among 1st year healtcare students). Ulusal viral hepatit kongresi kongre kitabi, viral hepatit le savasim dernegi. 2004:73.
46. Yegane-Tosun S, Ertekin E, Ansoy A, Ozbakkaloglu B. Saglik calisani adaylarinin HBV’den erken donemde korunmalarinin onemi (The importance of protection from HBV among healthcare workers). Ulusal viral hepatit kongresi kongre kitabi, viral hepatit le savasim dernegi. 2000:P-B20.
47. Tulumoğlu Ş, Uraz G, Türkmen Ş. Yaşları 20 – 60 arası olan 317 erkek mahkumda HBsAg, Anti-HBs ve anti- HCV prevlansı (HBsAg and anti-HCV seroprevalence in convicts between 20 – 60 years old). 29th Turkish Microbiology Congress Abstract Book. 2000:397.
48. Altındiş M. Afyon Cezaevi Mahkumlarında Hepatit B ve C virus enfeksiyon sıklığı (Hepatitis B and C prevalence in convicts in Afyon Prison). 5th National Viral Hepatitis Congress Abstract Book. 2000:P-B6.
49. Yegane-Tosun S, Erdurak K, Ertekin E. Mahkumlarda HBV ile karşılaşma durumu ve aşılamanın önemi (Preavalence of HBV exposure in convicts and importance of vaccination). 6th National Viral Hepatitis Congress Abstract Book. 2002:104.
50. Ozdemir R, Ozgul A, Gunes M. Izmir genelevinde calisan kadinlarin HbsAg, anti-HBs, anti-HIV, VDRL, TPHA yonunden degerlendirilmesi (HbsAg, anti-HBs, anti-HIV, VDRL and TPHA prevalence among female prostituse in Izmir). Turk klinik mikrobiyoloji Kongre Kitabi. 2002:353.
